# 

**Published:** 1847-02

**Authors:** 


*Buffalo Medical College*—The preliminary course of instruction in practical Anatomy in this institution is now in progress. The lecture session commences on the 24th inst. The building is in readiness, and the prospects highly gratifying.

